# Bibliographical Notices

**Published:** 1843-07-01

**Authors:** 


### Periscope;

OR,

### CIRCUMSPECTIVE REVIEW.

"Ore trahit quodcunque potest, atque addit acervo."

### Notices of some New Works.

ON SPASM, LANGUOR, PALSY, AND OTHER DISORDERS, TERMED NERVOUS, OF THE MUSCULAR SYSTEM. By *Arthur James Wilson,* M.D. Physician to St. George's Hospital, 8vo. pp. 200, Parker, Strand, 1843.

THIS is the product of a talented physician, who has (and who has not?) some peculiar opinions—especially respecting the blood. The little work is dedicated to the memory of his father, the late James Wilson, Esq. whose accurate anatomical knowledge will long be remembered by those who studied in Windmill Street, at the beginning of the present century.

Our author considers that the muscular fibre—"the ever busy self-moving fibre," has been too much overlooked in tracing the pathology of diseases, in the same way as the state of the blood was neglected. One cause of this neglect, he thinks is the fact that muscular structure does not present such evident changes after death, as glandular and other structures. The muscles, too, have been despised, as being entirely excited by the nerves supplying them.

"From within, as from without, from the air we breathe, from the food we swallow, as from the inmost recesses of the most distant structures, there is a channel by which, more directly than by its nerve, most of what influences the muscle is received into its fibre. It is by the continuous universal blood, which, while it bathes the fibre, touches the air and mixes with the food—it is by the blood in mass and in current, that the muscle maintains its great and constant relations with the external agencies of matter as with the elementary texture of every organ in the body.

"Let us, therefore, in the enquiry we propose, be content to approach the muscle with the material of its circulation, rather than from the distant sources of its so-called 'nervous energy.'

"In rational medicine, as in the physiology of health, the blood, we shall ever find, is our one helping clue. From the blood deficient, from the blood in excess in the muscular structure, a prejudice to its function invariably follows. Spasms, the most frightful, may be induced by the addition of certain principles to the blood; and from the absence of others, the faculty of self-contraction in the fleshy structures may be entirely suspended.

"In the explantion of such agencies of the blood upon the muscle, there is no need for the intervention of the nerve. The effects are direct upon the muscle; and, therefore, upon the nerve, as being one of the parts of its entire organized structure. States of the muscle are necessarily implied by states of the blood. And, upon the blood, let it be remembered, not only in the composition of its material, but upon the blood organized and discharging functions many and various; upon the blood in its 'habits as it lives,' the muscle is made to depend. Its lively function at once ceases under any great vice of the blood."

Our author goes on to say that, as the blood influences the muscle, so the muscle, in its re-agency, influences the blood. The muscle is not to be considered as merely the organ of motion, but "collectively and in the mass, as the most extensive of living structures," constantly acting upon and "elaborating the great common material of the body, and preparing it for circulation elsewhere." Dr. Wilson gives his definition of "MUSCLE" as "the living flesh, in its combination of many parts, with the blood predominant in all;" and by muscular action, he "never means less than a result of the triple agency of nerve and fibre with the blood." There is a depth—perhaps a certain degree of obscurity—in some of Dr. Wilson's reasonings or theories, which induces us to pass on to their application to practice or facts.

"In the practical application of these principles to the treatment of muscular disorder, we find that great advantages are actually obtained by addressing our remedies to the muscle through the wide current of the blood that pervades its entire texture, rather than by seeking to influence the structure through the exclusive agency of its nerve. Medicine and disease work, it is ever found, on the same living materials by the same means of living action; and thus it follows, that sometimes by extending the supply of blood to the muscle, sometimes by reducing it, by removing hurtful principles from the circulation, or by restoring those which are inherently wholesome, we do in truth best control the symptoms of muscular disorder."

The disorders, he observes, incidental to the voluntary muscles, are principally expressed by undue violence or contraction—by slowness or irregularity of movement—and by suspension of motion. Cases, he avers, of palsy and chronic spasm, "hopeless, if assumed to depend on organic disease of the brain or spinal marrow, are not unfrequently cured," by means addressed to the blood and its purification. In such cases, large bleedings ("the routine practice of the day") and "strong mercurial poison" are, of course, condemned.

"Even where the muscle is least in fault—when its function is impaired by lesion, not of its own structure, but of the brain or spinal marrow—even under these effects of distinct and distant nervous injury, we must in our management of the case still depend on the blood for all that we hope to secure of improvement in the muscle."

Our author makes several apt remarks on the state of the voluntary muscles in various diseases. Thus the poison of fever, through the medium of the blood, soon prostrates the strength, and affords important indications in the way of prognosis. The expression of the countenance, on which we so much rely, is formed entirely by the state of the muscles. "In the convalescence of fever, the restoration of the muscular power is the completion of recovery."

### SPASM.

This is, as Dr. W. observes, a vague term in medicine. It is applied to the involutary muscular fibres, as well as to the voluntary—nay, it is applied to structures where no muscular fibres have yet been demonstrated. Dr. W. however, applies the term "spasm" only to those muscles which are under the influence of the will.

"It is not, however, by organic disease of the brain, or by tumors pressing on the nerve, that spasm is for the most part explained. The worst forms of spasm seem to have least to do with the nerves of the affected muscles, and are compatible with the soundest state of their structure. Indeed, when spasm kills, we rarely learn, by dissection of the 'Nervous System,' how death is brought about.

"On the other hand, large portions of the brain or spinal marrow may be slowly disorganized with no effect of spasmodic contraction in those muscles of which the nerves are continuous with the damaged portion of the medullary column."

Spasmodic affections at the commencement of fever, are often indicative of an eruptive complaint, as all experienced practitioners must have observed.

Dr. W. confesses that he cannot easily account for cramp or true spasms: but, in his own practice, has always found it accompanied by some "constitutional disturbance." He will not admit the theory that cramp depends on "pressure or irritation of the nerve of the affected muscles," or from sympathy with other nerves.

"It is from what offends the blood in the fibre, that spasm of the voluntary muscles is for the most part induced. By no contrived irritation of the ganglionic nerves can we directly induce spasm in the voluntary muscles; let, however, but a few drops of the solution of extract of the nux vomica be injected into the current of the living blood, and tetanus is the certain result. There are no cramps so severe as those of which poison mixed with the blood is the direct cause. Can we refuse the inference, that always in the muscles, when thus disturbed, there is a prejudice of the blood by composition or in function;—and by the blood's function how much is implied!—how much more than the little we know of it by its material composition, or in its mechanical relations! This has not been enough considered. The blood not only lives, but its life predominates over that of all else in the system."

The sense of weight and constriction under the ensiform cartilage, together with the anxiety and other sensations during a paroxysm of "spasm in the chest" (we suppose Angina Pectoris) give evidence, our author thinks, that the blood is in undue volume, or of unfit quality in the large vessels.

But we have followed our author through a host of spasmodic affections, on which he descants, but so often in a mysterious language and phraseology, that we confess ourselves unable to fathom his meaning on many occasions. That he attributes the wide class of ailments, referred to in this volume, to some morbid condition of the blood, we think is clear; but what that morbid condition is, or how it is to be remedied or prevented, we have been unable to learn from the author himself. Clearer and abler heads than ours may, probably, derive sound precepts and important information from Dr. Wilson's volume, and to them we recommend its attentive perusal.

We shall give one more extract, and it is one of the least equivocal in the volume.

"The mind is educated with the muscle,—lives with it, and by it; and in the body never survives its death; but muscle is not therefore mind. The beasts that perish have muscles like ourselves. That in man the mind should conform with, and be dependent on matter, is the law of his probationary existence. As a compound being, on the Creator's design, he is fashioned, taught, and tried. To distinguish mind, in man, from matter, is to unmake the man.

"It is a common error with divines, and much scandal has arisen from the impossible attempt. Man's mind is not a necessary, or fortuitous result of his material life, but an imposed condition of its tenure. It is dependent, as we have seen, at all times, on the muscles for its expression. Mind, if anywhere in the body, is everywhere. How grossly do they err, who, fearing to acknowledge its close, constant, and entire connexion with every drop and particle of the living human frame, would limit its residence to certain parts, as to the brain!

"But, if thus localized in the brain; in which brain? for there are two; in which lobe, chamber, or convolution of the brain? for there are many. The brain is but the central union, by double junction, of all the nerves of all the muscles; an appended system of parts, with partial relations, by function, with other structures, and capable of common influences, only through the blood. It, the brain, may, in truth, be regarded as the lungs of the nerves; for, in the brain, the nerves are spread out, universally and simultaneously, to the blood, fresh, full, and ventilated from the aortic heart. Without the adjuncts of blood and muscle, we cannot get a notion of the brain, either in structure or function.

"Thought and Memory attach to the brain, only as representing the several organs of the body, in combination, by their nerves. There is no more thought in one part of the brain than in another; than in the muscle, whose nerves are there expanded, or in the blood, of which the brain in all its parts consists. On the triple relation of blood and brain, by nerve, with the fibre, the expression of thought is made, in the muscle, to depend; and of thought, in its physical operation, this is all that we are permitted to know.

"That the soul, by which we feel and think, is essentially, now, and for ever, distinct from blood, brain and muscle, we are assured by a Law, which is not of the flesh. And yet, by the flesh, is the law to us made manifest. It has been written, spoken, acted,—still, by those muscles which have been our theme, and from which we now with awe retire. That the Word might to Man be Light, it became itself incarnate."

---

Numerous Cases of Surgical Operations, without Pain, in the Mesmeric State; with Remarks upon the Opposition of many Members of the Royal Medical and Chirurgical Society, and others, to the reception of the inestimable blessings of Mesmerism. By *John Elliotson*, M.D. 8vo. pp. 93. Bailliere, 1843.

This pamphlet is generally denominated "Dr. Elliotson's farewell to the profession." We are sorry to see it, for it is probable that before Dr. E. dies he will regret its publication. We never heard any one deny the talents, abilities, and industry of the author; but genius is very often allied to eccentricity—and the latter not seldom borders on delusion. There is no doubt that Dr. E. firmly believes in the truth of Mesmerism; but he ought to recollect the maxim of Hippocrates—"Judicium difficile experientia fallax." If experience be often fallacious, how much more so is *experiment?* When the generality of mankind differ from an individual on some particular point, it is a strong presumption that the individual is wrong, and he had better give way. Instead of this prudent step, the Mesmerist immediately points to Galileo, Harvey, or Newton,—forgetting the hosts of enthusiasts who considered themselves Galileos, but whose doctrines are now consigned to oblivion with themselves. Galileos, Harveys, and Newtons, are not the products of every year—nor of every century, whereas visionaries are as plentiful as blackberries, and often as transitory as that sable fruit. But, say the Mesmerists, "we have disciples among the highest and most learned classes of society." As for the *higher* orders, they are ten times more gullible than the middle classes—and among the learned, there will always be found credulous and superstitious individuals. Johanna Southcote did not want *believers* among the better classes of society. Even Doctors asserted that they felt the young Shiloh skipping about in the womb of a sexagenarian and flatulent old hag.

There is no question, as we before said, that Dr. Elliotson is sincere in his faith; but how he can so far blind himself as to suppose that Mesmerism, *if true*, would be an inestimable blessing to mankind, we cannot comprehend! What! the power, (not limited to the faculty, but open to all,) of casting man, woman, or child. into such a miraculous trance that their legs may be amputated—their teeth drawn—needles pushed into their flesh—ammonia inhaled—their jaw-bones cut away—without the slightest consciousness on their parts!! Why such a power transcends that which was ever laid claim to, by any necromancer or magician that ever lived! If these faculties or powers were real, we ask Dr. Elliotson, what man's life or woman's virtue could be safe from the witcheries of the Mesmerist, in any part of the globe? Dr. Elliotson will, doubtless, answer, that the faculty and the public at large, have *now* arsenic, corrosive sublimate, and prussic acid, which they may convert into poisons, though generally used as remedies. Softly, good Sir! It is not quite so easy to thrust arsenic down a person's throat, as to lay him or her fast asleep by a few passes of the hand—whilst the act of poisoning is more easily detected than that of mesmerizing—and the penalty would be a swing at Newgate. Notwithstanding, the dreadful pains and penalties against poisoning, have we not daily examples of these crimes being practised, even at the risk and often the expense of the criminal's life?

And if the miraculous power of transferring vision to the epigastrium, audition to the patella, and a knowledge of distant events to a person sound asleep in Dr. Elliotson's library, can be effected by waving the hand over the face of an individual, in what utter contempt must we not hold the paltry miracles of Holy Writ?

But we must proceed to take a short notice of the pamphlet before us. Dr. Elliotson, like a prudent Chinese general, fires off his great gun at the commencement of the battle, in order to frighten the barbarians and put them to flight at once. This large piece of ordnance is levelled at the Medico-Chirurgical Society and its members, since a demolition of that body would clear the way for a triumphant march to the mesmeric corps. The style and taste displayed by Dr. Elliotson in this literary warfare, are anything but creditable to himself. But upon this point we will not trust ourselves to comment. We shall quote a passage from our contemporary, the PROVINCIAL MEDICAL JOURNAL, which may shew Dr. E. the opinions which are entertained on the subject in question.

"Not content with exposing fully to his own satisfaction, at least, the opposition to the doctrines of mesmerism, he fastens upon the speakers themselves, and leaves them not until he has told us that Dr. Johnson is a reviewer—that Messrs. Liston and Wakley are now inseparable friends—that Dr. Roots is a physician, living in Russell-square; and, not yet satiated, he animadverts alike upon his professional brethren, their pursuits, and their very houses; and hovering about for some farther object, he at length finds a climax in the 'two very large brass plates of the M.D. in Hill-street.'

"Weak indeed must be the argument that can need support such as this; every man must bring disgrace upon himself and the opinions he endeavours to uphold, by such discreditable means. Careless, as it would seem, alike to all claims of good feeling and good taste, and in apparent eagerness to make his attacks as unjust as vituperable, he hurls still direr weapons at the heads of those who have taken but little part in the discussion of his opinions. One of these he apostrophises as, 'Ungenerous man! I envy you not your heart!'—Are there no passages, we would ask Dr. Elliotson, in his book which will deservedly draw down upon himself, from the lips of every professional brother, the epithet he has thus unhesitatingly applied to another? Heedless of such reflections, and reckless of whom he attacks, the grave itself affords no shelter to its mute tenant; and Sir Astley Cooper is for the first time described as having been 'but scantily informed, and endowed with but a moderate degree of the higher intellectual faculties.'"*

---

* Provincial Medical Journal, April 29th, 1843.

The great gun, to which we have alluded, is, of course, the mesmeric amputation; and the truths of mesmerism are grounded, first, on the wonderful improvement which the patient experienced in the hospital, while being mesmerised—anterior to the operation—and, secondly, his insensibility to pain during the amputation itself. It is to be remembered that the patient was a labouring man, who had suffered from diseased knee-joint for five years, and was obliged to "earn his bread by the sweat of his brow," up to the time he was admitted into the hospital of WELLOW. Now, would any man but a mesmeriser fail to perceive that the comforts, quietude, and proper remedies of an hospital, would work an immense change in the general constitution of a patient, snatched from the plough-tail with diseased knee-joint, and placed in the ward of a public institution—independent of the pokings and pawings of Barrister Topham? Well! He was mesmerised daily—and perhaps "*something more*"—with improvement of general health. Here we ask the Barrister and his dupes, why he did not *cure* the disease of the knee-joint? Was it more difficult to anchylose that said joint, than to change an umbilicus into an eye, or an olecranon into an ear? But the mesmerisers well knew that it was far easier to induce a peasant to feign a sleep, than to feign the cure of a disease whose existence would still be patent to the world. They therefore determined to amputate. What was the mode of training for the operation we shall not determine. The surgeon, strange to say, was absent from his duty, and the Barrister had charge of the patient!! After no small number of mesmeric passes, the peasant assumed the appearance of coma, and underwent amputation—not, nowever, without evincing signs of the agony he suffered, or the greater agony of suppressing his sufferings! "A low *moaning* was heard at intervals until the conclusion of the operation?"

Dr. Elliotson appears to have adopted the maxim "de non *apparentibus*, et non *existentibus* eadam est ratio." Now, however this may hold good in physics—and even *there* it is not always to be relied on—it will prove a most erroneous dogma in morals or metaphysics. Where is the *appearance* of deadly hatred and determined revenge, in the gracious smile and loving embrace of the courtier? And yet the most diabolical passions are masked under the appearance of Heaven-born benevolence? Did the Prelate, who held his hand in the fire, till it was reduced to a cinder, as a punishment on the member for signing a bloody document, *feel* no pain, though he evinced no *sensation* of it? But even if Mesmerism did obliterate the sensation of pain in a surgical operation, we would not accept the boon. Nature has given to every created being that possesses a voice, a language to indicate pain, and the suppression of that language is injurious. Smothered or concealed feeling, either of pain or sorrow, is almost invariably inimical to healthy function.

Like most deceptions or impostures, the case of James Wombell proved a great deal too much, as was shewn by Dr. Marshall Hall, Sir B. Brodie, and others. The unamputated limb, if the man had been asleep, and consequently had no *voluntary* power over it, would have started the moment the incision was made through the skin, and still more strongly when the nerves were pinched. The deception, therefore, betrayed itself, and it is clear that neither the operators nor the patient had studied the "reflex function."

The great gun, then, being proved to have had no shot, but merely wadding, we shall take no further notice of it. The greater portion of this pamphlet is taken up with comments on the Medico-Chirurgical Society, and such of its members as took part in the discussion. These remarks we shall leave untouched. Dr. Elliotson will, one day, regret that he has given way to his feelings, and indulged in personalities, which always damage a philosophical argument.

Some of the minor proofs of Mesmeric truth are not a little curious. Mrs. Valiant's nursery-maid figures among these. She was prepared for a painful operation by Sir Thomas Willshire. In six minutes she was asleep, and could *not* hear the loudest noises; but when Sir Thomas took her hand and spoke in a

*low voice*, she *heard*, and answered that SHE WAS ASLEEP AND COMFORTABLE" ! ! ! "She opened her mouth for the operation to be performed at the command of the Mesmeriser." "Sir T. took some wine, while the patient went through the form of tasting and drinking; and, on being questioned, said she tasted wine:—the same experiment was tried with a piece of biscuit, and she said she tasted the biscuit." All this, too, when in such a profound Mesmeric coma as to have her jaw cut away without feeling the operation ! ! !

When such transcendental absurdities are published as proofs of Mesmeric truths, we give up all argument in despair. We might as well expostulate with the waves that chafe upon the solid rocks or idle sands. Dr. Elliotson remonstrates feelingly against the uncharitable and unchristian practice of *suspecting* poor innocent females of deception. Alas! the history of mankind presents little else than a tissue of deceptions and crimes. We wish the worthy Doctor was a little more suspicious himself! At the same time, we freely admit that HE is, beyond all comparison, the most honest and the most talented of the whole sect of Mesmerisers. But no man is without a weak point—and unfortunately where there is a weak point, *that* is considered by the owner as the most impregnable portion of his intellectual or moral system. We have not the slightest doubt that Dr. Elliotson as firmly and sincerely believes in clairvoyance and transposition of the senses, as the Pope believes in transubstantiation—the Jew in Exodus—the Christian in the Crucifixion—the Turk in the Paradise of Houris—or the Hindoo in VISHNA and SEEVA.

---

### THE AMERICAN JOURNAL AND LIBRARY OF DENTAL SCIENCE. New York and Baltimore.

WE have received the first Number of the second Volume of this quarterly Journal published in New York and Baltimore, under the auspices of the American Society of Dental Surgeons—"the only regularly organised and extensive association of dental surgeons in the world." Our Transatlantic brethren have fairly got the start of the Old Country, and we may say of all Europe, in this respect, and much praise we give them for their energy and zeal. We cannot indeed wonder much at this, after reading the soul-stirring oration of Dr. *Solyman Brown*, at their last annual meeting, on the Pursuit of Professional Eminence. It may be a little too magniloquent in parts for our phlegmatic tastes; but where is the man whose ambition and thirst for fame will not be kindled by a fervid appeal to the names of Columbus, Milton, Cicero, Buonaparte, Wesley, and Washington, he "who unfurled the banner of universal freedom on the castellated battlements of the world!" The writer points out with great force of illustration the necessity of a man, if he aims at distinction in dental surgery or indeed in any pursuit whatever, devoting himself to that sphere of life for which he is best suited by his natural qualifications. "Let every individual," says he, who indulges the ambition of rising to professional distinction, see well to it that he is not a Mercury attempting to handle the thunder-bolts of Jupiter; for although the former was one of the accredited Gods of Olympus, when he burned his fingers (!) he was meddling imprudently with the attributes of another."

Scarcely less eloquent is the opening address of the President of the Society, Professor *Hayden*. We wish much that we could spare room at least for the peroration; but perhaps it is just as well that we cannot; as the unsatisfied curiosity of our readers may induce them to purchase the Journal for themselves.

From the second article in this number, a Dissertation on the Management of the Mouth and Teeth, by *L. S. Parmly*, M.D. D.D.S. (we suppose these last initials stand for Doctor of Dental Surgery) we select the following two extracts, as practically interesting:—

"There is no truth better understood, and there is none which every day's observation more clearly proves to medical men, than that the loss of teeth is greatly augmented since the introduction of knives and forks, by which the daily friction upon the enamel has been lessened."

How far medical men in this country may approve of the following directions, we shall not take upon ourselves to determine:—

"As soon as the teeth (of an infant) make their appearance, it should be the duty of the nurse or mother to clean them morning and evening with a small brush and water; and, as they increase in number, floss-silk, flax, or hemp well waxed* should be regularly passed between them, moving it up and down a little under the gum, not only for the purpose of removing accumulations (surely these must occur unusually soon in American infants), but also to give a fine polish to the enamel, which is the natural protection of the bone and beauty of the teeth."

We most cordially wish every possible success to our enterprising *confreres* on the other side of the Atlantic, and will always be most happy to notice their labours when submitted to our review.

---

De l'Idiotie chez les Enfans, &c. Par *F. Voisin*. Octavo, pp. 122. Paris, 1843. Bailliere.

This brochure contains eight articles (most of them of old date and now republished) on several questions connected with insanity—using that word in its widest acceptation. There is exceedingly little novel or instructive matter in any of them; and we shall not therefore occupy space, that can be more profitably employed, with an analysis of their contents. M. *Voisin* is an ardent phrenologist, and his writings are imbued with a strong bias to examine all the varieties of mental aberration on cranioscopic principles. He seems to be of opinion, that in the greater number of criminals the brain is badly developed; for he tells us that out of five hundred young prisoners whom he had inspected, "two-thirds, of them, so far from being liberally endowed by Nature, have been more or less disgraced (disgraciés) by her, and are suffering the penalty of an incomplete organisation. Without being reduced to an intellectual, or, properly speaking, a moral idiotcy, they are unquestionably below the average standard of organization, and exhibit very distinctly the impress of their mutilations. The brain in these persons is at the *minimum* of development in its anterior and superior portions—those very portions which make us what we are, and raise us above the brutes."

We dislike the spirit of such language as this. That the intellectual and moral endowments of different men differ not a little, quite independently of education, we are ready to admit; but we can never bring our minds to allow that the majority of criminals are so irresponsible for their actions as the phrenological doctors would wish us to believe. What think our readers of the following passage? "May not a person have more or less intelligence, and yet be affected with an idiotcy in his moral sentiments? There are not wanting many examples of individuals who, either from a *vice* of nature or of education,

---

* In a subsequent note we are informed that these and all other materials requisite for a dentist are kept for sale by Dr. *Solyman Brown* of New York, the co-editor of the Journal.

seem to be utterly destitute of all goodness, justice, nobleness, and veneration. With regret I must acknowledge that even in our social hierarchy there are several characters who appear to be only intelligent (not moral) animals." This doctrine, it seems to us, may be said to contain about one grain of truth in a bushel of error. But let us not shut our eyes to the good points in M. *Voisin's* reasonings: witness this extract.

"It is by working in this direction, by overstepping those lines of demarcation which pride and ignorance have set up between man and man, by studying the brains of individuals and of nations, by disclosing their organisation, by shewing that they are all made upon one model, by placing on the same line, as to their intellectual and moral faculties, alike the Negro and the European, that science may yet have to rejoice in giving a firm basis to morality, or at least in consecrating some of its best principles. It justifies the eloquent pleadings of those gifted men who, being far a-head of the age in which they lived, have deemed it their sacred duty to consider the whole human race as one family, and to claim for every one alike equal liberty, justice, and protection."

---

ELEMENTS OF GENERAL PATHOLOGY. By the late *John Fletcher*, M.D. Edited by *John J. Drysdale*, M.D., and *John R. Russell*, M.D.

WE gave some account of the two first divisions of this work in the 74th number of this Journal, and we now proceed to offer a few remarks on the third and last, or therapeutical part. This need not detain us long; for it is very meagre, and contains scarcely anything of novelty or of practical importance. Dr. Fletcher had evidently but little experience in the practice of medicine. The shortness of his professional life, and his multifarious engagements as a teacher of anatomy, physiology, &c., precluded him from that intercourse with the bed-side of the sick, which is essential to a proper appreciation of the effects of remedies, or to their adaptation to the various symptoms and shades of disease. In former ages no subject relating to medicine occasioned such constant warfare as the question whether the practice of physic should be founded on principles of reasoning, or on experience alone. The good sense however of modern times has shewn, that to form the accomplished and successful prescriber, it is necessary that scientific research and inductive reasoning must go hand-in-hand with observation and experience.

The great attention that has of late years been paid to the study of morbid anatomy and pathology, has wonderfully advanced the science of medicine, and rescued it from the stigma of empiricism formerly attached to it. In no department of science has there been greater progress made than in that of physiology and pathology. The functions of the living body when in health, or when influenced by disease, as well as the products of this diseased action, are now well understood, and we are indebted to the author of this work and to similar writers for much of our information on these subjects. We apprehend however that a knowledge of physiology and of disease—its symptoms and its consequences—has advanced with more rapid strides, than (what should be the aim and end of all medical enquiries) an acquaintance either with the means of prevention, or of cure. Whether the examination of morbid lesions and structural changes after death, by demonstrating their incurability, tends to diminish confidence in the efficacy of medicine, or whether the art of prescribing be held subsidiary to that of prognosis and diagnosis, there is but little doubt that the most accomplished physiologists and pathologists have not been always the most successful practitioners; and that we are indebted to observation, or perhaps to chance, rather than to pathology, for most of our successful therapeutical agents. Dr. Baillie, whose intimate and profound knowledge of the functions of the human body, both in their healthy and morbid state, enabled him to discriminate disease, and to foretel the result, with a precision before unknown, was said to be not fertile in his remedial resources. The science which taught him to predict the event, shewed also the inefficiency of human means to avert it, and thus perhaps, in some degree, paralysed his efforts; feeling that nothing effectual could be done, he did nothing.

The facilities which are afforded on the Continent for the prosecution of morbid anatomy, and the zeal with which it is studied, have advanced with very rapid steps the science of pathology; but, as in this country, we much question whether the cure of diseases has made similar progress. Indeed, on reading the works of foreign medical writers, particularly the French, one cannot fail to be struck with the accurate detail of symptoms—the clear, distinct, and true diagnosis and prognosis—and the general inertness and inefficiency of the remedial measures. Diseases seem to be left to run their course to display the diagnostic skill of the practitioner, and to furnish materials for the morbid anatomist and pathologist.

What we contend for is simply this—that an exclusive direction of the mind to anatomical and pathological pursuits, has a tendency to lead, and does in fact lead, to routine and inert practice. In proof of this we may refer to the works of many of our best pathological writers. Indeed nothing can illustrate our meaning more strongly than the therapeutical part of Dr. Fletcher's book; for, whether we regard the prophylactic or remedial measures, it is below mediocrity. It exhibits a sad contrast to the former parts of the work, which we had before great pleasure in recommending to the notice of our readers.

In explaining the mode of action of medicines in general, Dr. Fletcher has recourse to the same theory by which he accounts for the operation of morbific agents, viz. "*the specific nature of their stimulus.*" "It has," he says, "been sufficiently shewn that every organ of the body has a peculiar kind of irritability, adapting it to be acted upon by certain stimuli more remarkably than by others: and that it is owing to this peculiar susceptibility in certain organs, of certain impressions, that particular exciting causes, *e. g.* contagions, however applied, produce always particular diseases; and precisely the same explanation must be given of the more or less specific action of all medicines. Not only every class of medicines, but every individual medicine may be with great reason presumed, like every other agent, whether salutary or deleterious, to afford a stimulus more or less distinct from that afforded by every other. When received into the body therefore in any way, each will be comparatively inert with respect to all those organs to the peculiar irritability of which this stimulus is not adapted, and will act only or chiefly on those which are calculated to feel it, and this equally whether the medicine operate directly on the latter, or applied to some other organ, produces there such an irritation as, being conveyed by certain nerves to the organ upon which it is more properly to act, brings about its specific effects, in other words, operates by sympathy."

It is scarcely necessary to point out the inconclusiveness of this kind of reasoning, or its inadequacy to explain the operation of many of our best remedial agents. It is also in opposition to those doctrines of the humoral pathology which it has been the object of many of our best recent writers to establish on a more sound basis, and which explain much more rationally and satisfactorily the action of medicines. We took occasion, in our notice of the former part of this work, to object to some of our author's theories as to the cause of gout and other constitutional ailments; and the exceptions we then made will apply here. In carrying out his favourite notion, that irritation and sympathy are the almost universal causes of disease. He can see no other effect of medicine than that of producing irritation, and in this sense revulsion.

His views assimilate very much with those of Hahnemann, and, from the long and explanatory note of the principles of homœopathy, which the editors have appended to the text, we are led to infer that they have taken up, and cultivate this modern system of medicine. They have also given in a note, an account of the "wasser cur," or hydropathy, as it has been waggishly termed. This still more recent and simple method of treating disease seems, from its present popularity, likely to supersede the more mysterious system of homœopathy: and will probably continue in vogue until some individuals of consequence fall victims to it; or it be in its turn supplanted by some other vagary.

---

OBSERVATIONS ON THE EXTRACTION OF TEETH. With Plates. By J. *Chitty Clendon*, Surgeon-Dentist. 12mo. pp. 80. Four Plates.

THE objects of Mr. Clendon, an able and successful surgeon-dentist, are best explained in his own words:—

"Although many works on the Teeth—characterized by various degrees of excellence—have issued from the press during the last twenty years, I am not aware of any having a distinct reference to the branch of Dental Surgery, which forms the subject of the present treatise.

"In the works above alluded to, the natural history and diseases of the teeth—the preventive and curative means usually employed—the various operations which diseases in those organs render necessary—and even the most approved methods of supplying the deficiencies they occasion, are all dwelt upon and generally treated with great ability. But, however, serviceable these records of experience and research may prove to those who make this department their sole study and occupation, they are far too elaborate, and embrace too great a variety of subjects to interest, or be read by nineteen-twentieths of those on whom the operation of extraction usually devolves, and who—in reference to the teeth—seldom or ever perform any other. The consequence is, that while in every branch of the profession, both surgical and mechanical, which comes under the notice of the professed dentist, great progress and improvement have been made, the extraction of the teeth—the most frequent and painful of all operations—as far as the bulk of practitioners is concerned, has remained stationary,—the same errors, the same prejudices, and I fear I must add, occasionally the same unfortunate results, that formerly distinguished it, still, continuing to attend its performance.

"To supply this defect and remedy these evils, by submitting to the consideration of the numerous class of practitioners I have referred to, in a plain and distinct form, a knowledge of the safest and most approved method of performing the operation—a knowledge which is the result of a careful consideration of the subject, and confirmed by experience,—is the object of the present work."

And a very laudable and disinterested object it is—placing as it does in the hands of the profession those mechanical means and appliances which a more selfish spirit would have sought to confine to the individual, or his class.

Mr. Clendon thinks, of the key instrument, that it is often *difficult* of *application, unscientific* and *uncertain* in its operation, and always productive of *additional pain* and *subsequent inconvenience.*

Now as the key instrument is the one in most general use by practitioners, we think it would be no bad thing to allow Mr. Clendon to state the main objections to it.

*First,* he shews, as every body who has drawn teeth with it knows, that it is difficult of application.

"Before the instrument is applied to the mouth, it is first of all necessary to examine the tooth attentively, in order to determine the size of the claw to be used, the side of the tooth on which it will secure the firmest hold; and the space required between the edge of the claw and the fulcrum, so that they may act in a line as nearly parallel to each other as the principle of a lever will admit of. The two former points may be disposed of without much difficulty, but to decide the latter the nicest judgment will be required, for if the space in the *least* exceed the width of the tooth, directly the instrument is brought into operation, the fulcrum will inevitably travel from its position in an increased degree, and thus occasion loss of power; while the pressure and risk of breaking off the crown of the tooth, or fracturing the socket, will be considerably increased. On the other hand, should the space not prove sufficiently wide, the edge of the claw will be far distant from the line horizontal to the fulcrum; the effect will therefore be the same as if the latter had slipped below its proper position. To withdraw it from the mouth, to increase or diminish the space and re-adjust the instrument, under either of the above circumstances, is now the most proper course; but to do so would occupy very critical time and shake the confidence and perhaps the resolution of the patient—the original error, although by far the greater of the two evils, is therefore persevered in.

"The difficulty of forming a correct estimate of the space required, is sometimes increased by the tumefaction of the gum, occasioned by alveolar abscess, or gumboil, occurring near or on the very spot the fulcrum should occupy; to place the instrument properly it is necessary to allow for this, yet the moment pressure is applied, the space sensibly diminishes, and the fulcrum slips in proportion."

Frequently, the face and mouth are so inflamed and swollen, that the latter cannot be opened sufficiently for an accurate examination: so sound teeth have been removed for bad ones.

*Secondly*, the action of the claw is unscientific.

"The action of the claw was no doubt intended to *raise* the tooth from the socket, and most persons who use the instrument persuade themselves that this effect is produced;—a little reflection will very soon show the error of such conclusion. To lift a tooth without doing violence to the socket in which it is placed, the necessary extractive force must be applied either in a *perpendicular* or in an *oblique* direction: the want of sufficient space and risk of injury to the antagonist teeth will generally prevent the former, and to accomplish the latter, the sweep taken must be in proportion to the length of the roots and their position in the socket. Now the edge of the claw in turning describes the segment of a circle whose diameter is far too small to admit of the tooth being raised obliquely: the moment pressure is applied to the handle, the fulcrum turns abruptly on its bearing, and drags the claw in a parallel direction, the effect of which is to cant or upset the tooth—the claw itself preventing the former from rising over the edge of the socket.

"By removing the tooth in this lateral direction, the *natural* obstructions are very much increased, and from causes which I will presently explain, although they will perhaps be more readily understood by referring to the diagrams.

"If we suppose the crown strong enough to sustain a force adequate to remove the tooth, the claw in the first instance will press it against that part of the alveolus on which the fulcrum rests, while the latter, by its own pressure, in a great measure counteracts the effect produced by the claw, as it not only forces the alveolus against the roots, but prevents the former from yielding so as to afford a passage for the escape of the tooth; at the same time the extremities of the root press on the opposite side of the alveolus which offers an effectual resistance, and it is only when the fulcrum has turned sufficiently to allow the socket to break away, that the tooth can possibly be removed. When the latter is very firmly fixed, and the crown proves unequal to bear this lateral pressure, it will assuredly break off at the neck, a consequence the more likely to ensue, as the claw, however low it may be thrust, has a tendency, from the motion of the instrument, to rise until it catches the ridge formed by the terminal line of the enamel."

"As the teeth, from their peculiar structure and from disease, are more or less brittle, and will not bend to the sweep of the claw, they are consequently very liable to be broken;—and in those cases, where the crown is strong enough to sustain a pressure which will overcome the resistance of the roots, the socket must necessarily break away before the tooth can follow in the direction of the claw. Hence it is, on extracting with the key a portion of the alveolus is so often found attached to the side of the tooth next the fulcrum."

The fracture may extend to the neighbouring alveoli, and subsequent exfoliation be the consequence.

*Thirdly.*—The effects of the claw are uncertain. From the irregular form of the *neck*, the claw is exceedingly liable to *slip off the tooth* on either side. This objection will apply more or less to every tooth for the removal of which the instrument is commonly employed; but it will be most readily exemplified in the first and second molares of the upper jaw.

"The tendency to slip is also much increased by the free lateral motion of the claw, a space being left even in the best made instruments—perhaps intentionally—to admit claws of different sizes, which allows it to swerve both right and left. If the use of the key could not be dispensed with, an alteration ought at least to be made in this particular, and instead of allowing space for play, checks or guides should project from the shaft to keep the claw quite steady, and prevent it working out of a true line."

We say nothing of the pain produced by the pressure of the fulcrum—nor of obstruction caused by the irregular form of the roots—suffice it that Mr. Clendon believes that the days of the key-instrument are numbered.

The *Forceps*, then, is *the* instrument. Mr. Clendon deprecates their clumsiness and unsuitableness for the purpose they are put to. He observes:—

"In our own day a few practitioners, condemning the application of the key, have extracted *entirely* with forceps, and acquired just reputation and extensive practice by the facility with which they use them. Induced by these considerations, others have made trial and found them fail; they were more unsuccessful in their attempts than they had hitherto been with the key, and consequently returned to the latter instrument with increased partiality. This result might have been foreseen and is easily accounted for; the forceps *they* used were as unsuitable for the purposes to which they were applied, as common pliars or carpenter's pincers.

"As far as I have had an opportunity of judging, forceps have hitherto been made on most erroneous principles, and, generally speaking, provided they were large or small, straight or curved, was all that seemed requisite;—to adapt them to the various forms and positions of the teeth no one appeared to consider of any importance; in many instances the edges of the instrument clasped the tooth at right angles, and on bringing together the widely extended handles, one-fourth of the effort necessary to raise the tooth, converted them into a pair of nippers, which excised the crown in the most scientific manner. In some, the edges, although crescent shaped, were only half the breadth of the tooth—the extremities of the edge were therefore the only parts in contact; in others, for the front teeth, the mouth being intersected by a longitudinal groove, only a small portion of the edge, often a mere point, could press on the neck of the tooth; it consequently slipped long before the former showed a disposition to leave the socket. Others again were so unsuitable in form, that the blades actually crushed the body of the tooth before the edges came in contact with the neck; the joints were single, and entirely dependent, like scissors, on an indifferent rivet for support; this, and the great distance from the joint to the mouth of the instrument, rendered them liable to spring from the tooth the moment adequate force was applied."

Mr. Clendon obligingly offers to shew his forceps to any member of the profession who will call upon him. They have been constructed by Mr. Evrard.

We have some capital remarks on the Form and Construction of Forceps. We can only find room for the following :—

"Forceps should be made with the *mouth* adapted, in every instance, to the *neck of the tooth* to which it is intended to be applied: and when fixed, the former to be, as nearly as possible, in a line parallel with the root of the tooth; the edges sharp enough to separate the gum from the neck, yet sufficiently strong and well tempered to prevent their flying when force is used, and so fashioned as to encircle as much of the neck, as the proximity of the adjoining teeth will allow. The blades should be hollowed to suit the form of, and—to obstruct the view as little as possible—but just large enough to admit, the body of the tooth; —the joint double; the blades, to insure their steadiness, working within each other, and the distance between the rivet and the edges not greater than the length from the surface to the neck of the tooth will require; the handles particularly rough, not curved as they generally are, but nearly straight, and so close to each other, that when open and the instrument fixed on a tooth, they may not be wider apart than the hand can conveniently grasp; this is a point to which the greatest importance should be attached, for when widely extended they require a considerable effort to keep them together, which distracts the attention, and from the difficulty of regulating the pressure under such circumstances, the tooth is exceedingly liable to be broken off. The entire length of the instrument ought not to exceed six inches, with the exception of those used for the wisdom teeth, which, from their remote situation, require them a little longer.

"Forceps should in every respect be firmly and substantially made—otherwise the springing of the instrument might be readily mistaken for the yielding of the tooth, and such erroneous conclusion lead to difficulty and disappointment; a certainty on this point gives confidence to the operator, and instead of watching if the instrument maintain the proper position, he is enabled to direct his whole attention to one object, namely—detaching the tooth from the socket.

"The teeth, with reference to their removal, may be divided into eight distinct classes, requiring as many pairs of forceps, differing either in form or size, to correspond with the necks of the respective teeth. They are as follows :—

*In the Upper Jaw.*

1. The lateral incisors and bicuspides.
2. Central incisors and canines.
3. First and second molares, right side.
4. The same, left side.

*In the Lower Jaw.*

5. The incisors and canines.
6. The bicuspides.
7. First and second molares, right and left side.
8. Dentes sapientiæ, or wisdom teeth of the upper and lower jaws."

We must refer to the work itself, for ample directions with regard to the application of the forceps to individual teeth. This is indispensably necessary for success. Mr. Clendon recommends that the names of the teeth for which the several pairs of forceps are intended should be engraved on the handles of the instrument, which may be done by a weak mineral acid.

A word or two on the *Extraction of the Temporary Teeth.*

As a general rule, *the temporary teeth ought never to be removed until the roots are entirely absorbed, and their permanent successors sufficiently advanced to occupy the vacant places.* The spontaneous loosening of the tooth affords the evidence of this. Mr. Clendon thinks it of unquestionable importance to keep the temporary teeth in their places as long as possible.

"Many cases of permanent irregularity or crowding of the teeth, I am persuaded, are caused by the injudicious interference of persons, who, if required to examine the mouth of a child,—either from ignorance, or a still more culpable motive, always find it necessary to do something—the plea invariably being to *make room* for the new teeth, which, if time were afforded, would not fail, in nineteen cases out of twenty, to provide adequate room for themselves. Thus it frequently happens, from the increased size and early development of the upper and lower permanent incisors, the jaw has not *yet* expanded sufficiently to allow of their advancing in an even direction; the temporary canines are therefore removed without further delay or deliberation. Now, before these teeth are replaced, five or six years will probably elapse, and when at length they make their appearance, their places are already occupied by the bicuspides, which must in turn be sacrificed to make room for them. In this manner, and upon such pretexts, tooth after tooth is removed, and the size of the maxillary arch diminished; and that which was only a passing defect, ultimately becomes a permanent evil. Nor are such persons only to blame: many parents are so extremely anxious for the present appearance of their children's teeth, that they cannot possibly afford nature an opportunity of completing the work already commenced—they wish the impediment to be removed at once; and if the operator suggest delay, to see what improvement time will effect, he will probably be thought indifferent to his duty, and the child transferred to the *care* of those who would indulge no such inconvenient scruples, but comply without a moment's hesitation."

He thinks that "there are only two occasions which will justify the early extraction of these teeth: one is, extensive decay, producing frequent and severe pain, whereby the little sufferer's health or comfort is seriously affected, and which the usual applications fail to relieve;—the other, when the permanent tooth, already through the gum, takes up a position immediately behind the corresponding temporary tooth, the latter still continuing firm, and evincing no signs of a spontaneous removal. It should, however, be recollected, that as the presence of a full complement of teeth is necessary for the perfect expansion of the arch in which they are placed, their early removal is in either case an evil, and ought not to be effected until other means have been unsuccessfully tried, or until a reasonable hope that the defect will remedy itself, no longer remains."

Mr. Clendon sets forth at full length the advantages of the forceps. We need not, however, particularise what most scientific dentists are now pretty well agreed on. But, perhaps, a few of his *Hints to Operators* may not be unacceptable.

"To extract teeth skilfully, in addition to suitable instruments, *confidence* on the part of the operator is an indispensable qualification. On examining the tooth to be removed, he should feel convinced of success, and take every means to ensure it: if he have any doubt of the result, or of his own ability, the chances are the operation will be ill performed. Whatever he considers it expedient to do, should be done effectually;—half measures will never succeed—they only lead to vexation and disappointment. To use extractive force, for instance, when he doubts the position of the instrument, or the security of his hold on the tooth, is worse than useless;—it inflicts additional pain, and perhaps prevents a repetition of the attempt. The object should be, to place the instrument on the part experience points out as the most eligible; and until this is satisfactorily accomplished, no pressure should be applied.

"If the patient prove restless or refractory, it is better to desist at once, and wait until he is reassured; to persevere in a struggle, or to seize a tooth suddenly, with the intention of removing it at all hazards, is very bad practice, and ought not, under any circumstances to be attempted. Nothing tends so effectually to tranquillize the patient as a firm and quiet demeanour on the part of the operator; it will succeed much better than coaxing or expostulation, or than harsh and abrupt behaviour, which, especially at such a time, is very unjustifiable.

"Every thing in the shape of display, especially of instruments, should be carefully avoided; for, however the effect may impose on many, it only serves to excite the apprehension of the young and timid, and the ridicule, perhaps the disgust, of those who possess stronger nerves and greater discrimination. The place in which the operation is performed should not differ in appearance from other rooms, and the use of the formidable-looking chair dispensed with as much as possible. Under any circumstances, even during difficult and lengthened operations, a chair which will support and steady the patient's head is all that is necessary.*

"The operator should always place himself in front, or at the side, so as to face the tooth about to be removed. To stand on a chair behind the patient, or to seat the latter on the floor, with the head secured between the knees, are positions which should never be permitted, as they admit of undue violence, and have withal a most barbarous appearance.

"Some of these considerations may at first sight appear trivial to those who are accustomed to look upon the operation as an every-day event—in itself of little importance; but it should be recollected that a successful result can only be insured by attention to minute parts; besides, it is the patient—not the operator—who is to judge. However common-place the occurrence may be to the latter, to the former it is an affair of the greatest moment. At such a time nothing escapes attention—every little circumstance is noted, and produces an impression not easily effaced,—an impression that will certainly tell to the advantage or prejudice of the operator;—hence it is we daily find, that no distance, expense, nor even delay, will deter many from availing themselves of the services of those in whose reputed skill they place confidence. That some persons have more dexterity than others, must be admitted; but any one possessed of ordinary strength and an accurate knowledge of the part, by pursuing the methods I have pointed out, may remove a tooth without difficulty, and certainly without danger."

The length of our notice and quotations is a sufficient evidence of our good opinion of this little work. It ought to be in the hands of every medical practitioner, who, with his own, or with those of his assistants, ventures "to draw teeth."

---

### Medico-Legal Reflections on the Trial of Daniel M'Naughten.
By *J. G. Davy*, M.D.

We all know the violence and ferocity displayed by a part of the public press on the subject of M'Naughten's Trial. If ever a man laboured under monomania, M'Naughten did so, and if he was a responsible agent, nine-tenths of the inmates of lunatic asyla are most unjustly incarcerated.

The ignorance of those who affect to lead public opinion is gross in relation to insanity, and in proportion to their ignorance are their confidence and dogmatism. The clamour which they get up against the medical evidence and medical opinions, though laughable from its impertinence, is injurious in its effects,

---

* "It appears almost incredible, yet I have been assured of the fact by those, on whose testimony I can rely—and who mentioned it in terms of severe reprehension—that there are 'operating chairs' in use, with such a variety of movements and complication of machinery, as to resemble an improved portable crane, or some ingenious device for torture, rather than a chair for a person to sit on. It may possibly be suggested that they are intended to frighten away pain and supersede the necessity for extraction."

prejudicing, as it does, the community, and recoiling on the heads of the unhappy victims of law, legislation, and bigotry.

It is of paramount importance at the present moment to disseminate correct notions on the nature of insanity. The Judges are deliberating at the summons of the Chancellor, who, backed by an ex-Chancellor, himself a representative of panto-mania, seems far from disinclined to render the law of insanity stringent, and to hold all cheap but the judicial ipse dixit. That a set of men, whether clothed in red or black, who practically know nothing of the malady they pronounce upon, should settle ex cathedra what are its symptoms and its nature, is certainly absurd enough. That these same men should affect to look down on the opinions of those who make its investigation their business, and who, in all that relates to an acquaintance with the bodily and mental constitution, must be immeasurably their superiors, goes even beyond absurdity. But when the sum of their ignorance is to constitute the law, and become the Procrustian bed into which the wretched madman is in future to be forced, it is the duty of the humane and the enlightened, to oppose to the brutality of political rancour, and the assumptions of incompetent authority, the mild and steady dictates of science and of truth. Our course is clear. It is for us to state the facts as they are—to shew when and where and in what degree the mental powers are impaired—to disabuse the world of its vague notions of the *mind*—to point out the connection between mental and corporeal ailments—to explain the dependence of the former on the latter—and to establish the axiom that, as the brain is the instrument of the intellectual acts, it is to its alterations that we must look for the causes and the explanation of their aberrations. To whatever result the pursuit of truth may lead, it is immaterial to inquire. Fiat justitia, ruat cælum. That pursuit is the noblest accorded by Heaven to man, and it is a sort of blasphemy on Providence to pretend that the discovery of truth can be baneful. Society must be rotten when its parts are cemented by falsehood, and upheld by error.

We trust that our brethren will not be intimidated, by the blustering of any party, nor shrink from the fearless expression of what they think and know, in the Courts of Justice. A bold front and a calm disregard of bigoted or of interested clamour befits those upon whose evidence the life of the unfortunate maniac hangs. And whatever opposition may be made to the reception of truth, we may rest in the perfect confidence of its final triumph.

In the Work before us the insanity of M'Naughten is satisfactorily demonstrated. The various forms assumed by the disease, and particularly the several phases of monomaniacal delusion, are displayed by a reference to cases in the wards of the Hanwell Asylum, and the prevalent ignorance on the whole subject is conclusively exhibited and freely commented on. The author refers to the denunciations of the Press, of the *Times* especially, and takes that tone of humane indignation which might be expected from a man of science and candour.

